# Night shift work surrounding pregnancy and offspring risk of atopic disease

**DOI:** 10.1371/journal.pone.0231784

**Published:** 2020-04-16

**Authors:** Samantha Rada, Susanne Strohmaier, Aaron M. Drucker, A. Heather Eliassen, Eva S. Schernhammer

**Affiliations:** 1 Department of Epidemiology, Center for Public Health, Medical University of Vienna, Vienna, Austria; 2 Channing Division of Network Medicine, Brigham and Women’s Hospital and Harvard Medical School, Boston, Massachusetts, United States of America; 3 Division of Dermatology, Department of Medicine, University of Toronto, Toronto, Canada; 4 Department of Medicine, Women’s College Research Institute, Women’s College Hospital, Toronto, Canada; 5 Department of Epidemiology, Harvard T.H. Chan School of Public Health, Boston, Massachusetts, United States of America; Universita degli Studi di Milano-Bicocca Scuola di Medicina e Chirurgia, ITALY

## Abstract

**Background:**

Night shift work surrounding pregnancy may contribute to the risk of developing atopic diseases in offspring due to alterations in the prenatal environment, from stress.

**Objective:**

To examine the association of maternal night shift work surrounding pregnancy and offspring risk of developing atopic diseases from childhood to adolescence.

**Methods:**

We examined the association between night shift work before and during pregnancy among 4,044 mothers in the Nurses’ Health Study II (NHSII) and atopic dermatitis, asthma and hay fever risk in 4,813 of their offspring enrolled in the Growing Up Today Study (GUTS). Mothers reported whether GUTS participants had ever been diagnosed with atopic dermatitis, asthma or hay fever in the GUTS Mothers’ questionnaire. Generalized estimating equation regression models were used to estimate multivariable adjusted odds ratios (OR) and 95% confidence intervals (CIs).

**Results:**

There were no significant associations between pre-conception maternal night shift work and risk of atopic dermatitis, asthma or hay fever in their offspring. Among 545 mothers with information on night shift work during pregnancy, shift work also was not associated with atopic dermatitis, asthma or hay fever in the offspring. Stratified analyses by history of parental atopy and maternal chronotype showed some statistically significant findings, but they were inconsistent and no significant interaction was seen with increasing duration of night shift work.

**Conclusion:**

In this study, night shift work before and during pregnancy did not increase offspring risk of developing atopic dermatitis, asthma or hay fever.

## Introduction

Atopic diseases such as atopic dermatitis (AD), asthma and hay fever are among the most common chronic conditions in childhood and often coexist in the same individual [1,2]. Previous studies suggest there may be an “atopic march;” that these atopic manifestations progress sequentially beginning with AD and leading to asthma and/or hay fever [3,4]. AD is a common inflammatory skin disease that usually begins in childhood, with a prevalence of 20% in children worldwide [5]. AD can have detrimental effects on overall quality of life, whether the patient is a child or adult [6]. Among skin diseases, dermatitis is accountable for the largest global burden of disability-adjusted life years (DALYS) and morbidity (Years Lived with Disability) [7]. While AD is more prevalent in children under five years of age, current literature conveys that AD can persist into adolescence and adulthood [8–11]. It is important to understand this disease early on and how prenatal exposure to early life factors can potentially affect the onset and progression of this skin disease. Family history (e.g. shared genetics and environmental factors) has been mentioned as the most common driving factor for developing any atopic disease [1,12–16].

However, past research also suggested that changes in the prenatal environment, due to different maternal exposures [17–19], can influence the development of the fetus and potentially modulate the risk of diseases later in life [20].

Rotating night shift work is known to negatively impact circadian rhythms both in mothers working night shifts as well as in their unborn children [21,22]. It is considered an emerging risk factor for multiple chronic diseases and increased inflammation likely because of stress caused from circadian disruption [23–28].

In particular, stress due to night work appears to reduce oxidative DNA damage repair capacity due to melatonin suppression [29]. Previous studies have additionally reported epigenetic alterations in individuals who work night shifts [30,31], suggesting the potential for transgenerational impacts [32,33]. Further, the stress experienced from circadian rhythm changes during night shift work could potentially induce changes in the intrauterine environment [34,35], and is a potential risk factor for elevated immunoglobulin E (IgE) in cord blood, suggesting increased inflammation and transmission of IgE to the child, and T-helper 1 (Th1)/ T-helper 2 (Th2) imbalances in the in utero environment, all of which could enable the development of atopic diseases [36–38].

Little is known about prenatal occupational exposure as a risk factor for childhood and adolescent atopic diseases and there are no studies so far that specifically assessed the association of night shift work before and during pregnancy and the incidence of offspring atopic diseases. The aim of this study is to determine the association between maternal night shift work before pregnancy and during pregnancy and the risk of ever developing AD, asthma or hay fever in the offspring. We hypothesized that the risk of these atopic diseases in the offspring will be increased if mothers worked night shifts before pregnancy and that this risk would be even more elevated if mothers also worked night shifts during pregnancy. Because individual chronotype may interact with working times and thereby modulate disease risk [39], we tested how chronotype may modulate these associations.

## Methods

### Study population

The Nurses’ Health Study II (NHSII) is an ongoing prospective cohort study of female registered nurses that was established in 1989. A total of 116,429 nurses, aged 25 to 42 responded to a mailed questionnaire regarding their lifestyle, health behaviors and medical histories. Follow-up questionnaires are mailed biennially and used to update this information. Children, aged 9 to 15, who were born to women from the NHSII between 1987 and 1995, were invited to participate in the Growing Up Today Study (GUTS) in 2004. After obtaining maternal consent, invitation letters and questionnaires were mailed to 17,280 children which inquired about their health and lifestyle factors. Of these children, 10,918 completed and returned the questionnaires. Follow-up questionnaires, used to updated information on health and lifestyle, were mailed in 2006, 2008, 2011 and 2013. Mothers of GUTS participants also completed questionnaires related to their children in 2009.

### Exposure assessment

#### Maternal history of rotating night shift work before conception

Nurses were first asked to report the total number of years they worked rotating night shifts on the NHSII baseline questionnaire in 1989. Rotating night shift work was defined as working “at least three nights per month in addition to working days or evenings in the respective month”. This was updated in 1991 and 1993. The 2001 questionnaire retrospectively assessed night shift work duration for the time between 1993 and 1995. Since GUTS children were born up until 1995, baseline shift work history and updated shift work information was added together to derive a cumulative shift work exposure for shift work before conception from 1989 to 1995.

#### Maternal rotating night shift work during pregnancy

In 2001, NHSII participants who had reported one pregnancy since 1993 and were working as a nurse were sent a supplementary questionnaire which inquired about specific occupational exposures, including rotating shift work during their most recent pregnancy since 1993.

### Outcome assessment

The primary outcome was physician-diagnosed AD reported by the mother in the 2009 GUTS Mothers’ questionnaire, where mothers were asked if their child “ever had physician-diagnosed eczema (AD)”. As secondary outcomes we considered maternal reports of physician-diagnosed asthma and hay fever (also from 2009), since individuals with these atopic diseases are more likely to have a true AD diagnosis [2].

Additionally, we had information on child-reported AD outcomes from the GUTS questionnaires in 2006, 2008, 2013. These were combined with maternal reports of AD to obtain more consistent reports of AD diagnosis.

### Covariate assessment

The 2009 GUTS Mothers’ questionnaire assessed if mothers ever breastfed their child. Data regarding maternal and paternal physician-diagnosed eczema, asthma and hay fever was also obtained from this questionnaire. Parental atopy was derived by combining NHSII participant’s report of her own physician-diagnosed eczema, asthma or hay fever, as well as their child’s biological father’s diagnosis. Maternal age at delivery was calculated by computing the difference between mother’s and child’s dates of birth. Using the validated food frequency questionnaire (FFQ) from the 1991 main NHSII questionnaire, we were able to examine maternal diet quality, from which alcohol intake and the Alternative Healthy Eating Index (AHEI) was derived [40]. Energy expenditure in metabolic equivalent (MET) hours per week was derived from the 1989 baseline questionnaire when participants were asked to report frequency of various physical activities. Smoking habits and weight and height measurements before pregnancy were collected biennially and the information that was closest to, but before the conception of the first child included in the study, was used for determining smoking status and to calculate body mass index (BMI).

We obtained US Census tract data from 1989, including median household income and percentage of the population having a college degree in the neighborhood the mother lived, which was used to depict the family’s socioeconomic status (SES) around the child’s birth and early childhood. Other variables used to capture SES include family household income in 2001, husband’s level of education in 1999, and geographic region where GUTS children were living at baseline in 2004.

In 2009, maternal chronotype was assessed by a single question from the Morningness-Eveningness Questionnaire [41]. This question has been validated for classifying individuals as “definitely a morning type”, “rather more a morning than an evening type”, “rather more an evening than a morning type”, “definitely an evening type” or none of these [42].

### Analysis sample

The final sample for the analysis assessing pre-conception shift work and atopy outcomes included 4,044 mothers who had given birth to 4,813 children. Of the original cohort of 10,918 children, 4,720 were excluded because they were born before 1989 (the first exposure assessment of maternal rotating night shift work occurred in 1989). Further exclusions were twins and triplets (87 mothers, 195 children), non-full-term pregnancies (<37 weeks gestation) (1,001 mothers, 1,183 children), and mother-child pairs that had missing exposure information (6 mothers, 7 children), see S1 Fig for an overview.

The sample used to assess the association of shift work during pregnancy and atopy outcomes comprised of mothers who participated in the occupational supplemental questionnaire in 2001. A total of 621 mother-child pairs were identified. Of these pairs, non-full-term pregnancies (72 mother-child pairs) were excluded as well as pairs with missing exposure information (4 mother-child pairs). A total of 545 mother-child pairs were included in the final analysis of rotating shift work during pregnancy and atopic outcomes in offspring.

### Statistical analysis

Since siblings were included in the analysis relating maternal history of rotating night shift work before conception and atopic outcomes, we used generalized estimating equation (GEE) regression models to account for familial clustering in the estimation of odds ratios (ORs) and 95% confidence intervals (CIs) for AD, asthma and hay fever across four categories of cumulative rotating night shift work before conception (none, < 3years, 3–5 years, ≥ 6 years). We also examined these atopic outcomes in offspring by comparing mothers who ever worked rotating night shifts versus mothers who never worked rotating night shifts.

Basic models were adjusted for offspring age at baseline and sex. In multivariable model 1 (MV model 1), we further adjusted for maternal age at pregnancy, BMI and smoking status before pregnancy, AHEI diet score, physical activity, husband’s education level, parity, geographic region of residence at GUTS baseline, and the Census tract education rate in that region in 1989. Parental atopy was added to the existing covariates used in MV model 1 into our fully adjusted model (MV model 2). To test for possible effect modification, we assessed the association between night shift work and offspring atopic outcomes by including multiplicative interaction terms (night shift work and parental atopy; maternal chronotype) in multivariable regression models.

To obtain odds ratios and 95% CIs comparing children whose mothers worked night shifts during pregnancy to those whose mothers did not, we used standard multivariable logistic regression models with similar adjustment sets as described above.

All analyses were conducted using SAS version 9.4 (SAS Institute, Cary, NC) and all statistical tests were two-sided and considered statistically significant at p <0.05.

This study was approved by the Human Subjects Committees at the Brigham and Women’s Hospital and the Harvard T.H. Chan School of Public Health (Boston, Massachusetts).

## Results

### Maternal history of rotating night shift work before conception

The 4,044 mothers in our analysis were, on average, 33.4 (±3.6) years of age when they gave birth to the children in this study. The 4,813 children were, on average, 11.7 (± 1.2) years of age when they were enrolled in the GUTS study with 46.6% being girls and 53.4% being boys. A total of 2,620 mothers (64.8%) ever worked rotating night shifts. When comparing maternal characteristics, there were minor differences across categories of shift work duration (Table 1). Mothers who reported working a longer duration of shift work were slightly older at delivery and had a slightly higher BMI. They were also more likely to be past or current smokers before pregnancy and to adhere to the AHEI diet. Mothers reporting a longer duration of shift work also had higher rates of caesarean deliveries. Mothers who worked 3–5 years of night shift work had a higher frequency of AD and also reported a higher frequency of fathers (child’s biological father) having AD.

**Table 1:** Maternal and offspring characteristics by maternal rotating night shift work history before pregnancy, for 4,044 mothers and 4,813 children born between 1989 and 1995, enrolled in the growing up today study.

	History of rotating night shift work				
Characteristic	Never worked rotating night shifts	<3 yrs	3–5 yrs	≥6 yrs	Ever worked rotating night shifts
	(n = 1,424)	(n = 1,254)	(n = 946)	(n = 420)	(n = 2,620)
Maternal age at delivery, mean (SD)	33.1 (3.5)	33.2 (3.6)	33.4 (3.5)	35.0 (3.2)	33.6 (3.5)
BMI before pregnancy, ^a^ mean (SD)	22.7 (3.9)	22.7 (3.8)	23.0 (3.9)	23.3 (4.2)	22.9 (3.9)
AHEI,^b^ mean (SD)	42.6 (10.4)	43.5 (9.9)	43.7 (10.2)	44.3 (10.2)	43.7 (10.0)
Physical activity, MET-hrs /wk. ^b^, ^c^ median (IQR)	11.8 (4.9–24.8)	15.4 (6.9–30.3)	13.9 (5.6–29.7)	14.9 (5.4–28.4)	14.9 (6.1–30.1)
Smoking history before pregnancy, ^a^ % ^d^					
Never	75.3	74.0	73.2	63.2	71.9
Past	18.2	19.7	20.2	26.3	20.9
Current	6.5	6.4	6.7	10.5	7.2
Alcohol intake (gm/day) ^b^, mean (SD)	2.4 (4.7)	2.5 (4.8)	2.8 (5.1)	2.9 (4.5)	2.7 (4.9)
Husbands holding a graduate degree ^e^^,^ %	31.9	36.5	37.1	31.1	35.9
Household annual income category ^f^, mean (SD)	7.3 (1.7)	7.4 (1.7)	7.4 (1.7)	7.5 (1.7)	7.4 (1.7)
US Census tract median household income ^g^	62,644 (22,173)	63,142 (23,412)	62,942 (22,719)	61,272 (22,400)	62,779 (23,005)
US Census tract % college educated ^g^	30.6	32.1	33.2	31.6	32.4
Geographic region of residence ^h^, %					
West	20.5	14.8	12.9	10.1	13.4
Midwest	34.2	36.5	36.1	33.7	35.9
South	14.8	16.0	16.7	11.3	15.5
Northeast	30.6	32.7	34.3	44.8	35.2
Parity **before** first included pregnancy					
Nulliparous	20.3	21.8	22.0	20.5	21.6
One previous pregnancy	32.4	29.0	30.0	30.7	29.7
Two previous pregnancies	24.0	25.8	26.0	25.2	25.8
Three previous pregnancies	23.3	23.4	22.0	23.6	22.9
Breastfed ever^i^^,^^j^, %	86.5	88.7	87.9	84.2	87.7
Caesarean delivery, %	22.4	22.3	24.6	26.4	23.8
Mother ever had atopic dermatitis ^j^, %	8.6	8.2	9.9	8.6	8.9
Mother ever had asthma ^j^, %	11.2	13.0	11.5	12.6	12.4
Mother ever had hay fever ^j^, %	21.7	21.9	21.7	21.7	21.8
Father ever had atopic dermatitis ^g^, %	4.4	4.7	5.9	4.5	5.1
Father ever had asthma ^j^, %	8.6	6.3	7.5	8.1	7.0
Father ever had hay fever ^j^,%	20.9	20.6	17.3	17.1	18.9
Mother’s chronotype, %					
Definite morning type	31.9	36.0	34.4	37.4	35.6
Intermediate type	56.9	55.0	55.6	52.1	54.7
Definite evening type	11.3	9.1	10.1	10.5	9.6
**Number of pregnancies, n**	1,683	1,507	1,132	491	3,130
Offspring gender					
Male, %	46.1	45.0	48.2	49.3	46.8
Female, %	54.0	55.0	51.8	50.7	53.2
Offspring age at GUTS baseline 2004	11.8 (1.2)	11.6 (1.2)	11.6 (1.2)	11.6 (1.3)	11.6 (1.2)

Abbreviations: IQR, interquartile range; SD, standard deviation; AHEI, alternative healthy eating index; METS, metabolic-equivalent hours

^a^ Recorded on the most recent questionnaire prior to conception of first included offspring

^b^ Recorded in 1991

^c^ One metabolic-equivalent-hour is proportional to the amount of energy spent sitting quietly for one hour.

^d^ Percentages are of non-missing values.

^e^ Recorded in 1999

^f^ Reported in 2001, the scale had 9 levels, from 1 = <$15,000 to 9 = ≥$150,000

^g^ Recorded in 1989

^h^ At GUTS baseline (2004) West: AK,HI,WA,OR,CA,NV,NM,ID,UT,AZ,MT,WY,CO; Midwest: MN,IA,NE,KS,OK,WI,IL,IN,MI,OH,ND,SD,MO; South: AL,AR,GA,FL,MS,LA,TX,TN,KY,NC,SC,VA,WV; Northeast: DC,DE,MD,CT,NJ,NY,NH,PA,ME,MA,VT,RI

^i^ Only parous women

^j^ Recorded in 2009 GUTS Mothers’ questionnaire

Since differences between the age- and sex-adjusted versus multivariable models were small, we focused on the results from our fully-adjusted models (MV models 2). There was no overall association between maternal night shift work before pregnancy and maternal-reported offspring AD (OR_ever nightshifts_ = 0.98; 95% CI = 0.80–1.19) (Table 2). Shift work was also not associated with either asthma (OR_ever nightshifts_ = 1.09; 95% CI = 0.92–1.31) or hay fever (OR_ever nightshifts_ = 1.07; 95% CI = 0.89–1.28). There was no significant trend with increasing years of rotating night shift work (all P_trend_ > 0.05).

**Table 2:** Maternal report of child’s atopic dermatitis, asthma and hay fever: Adjusted odds ratios (OR) for offspring risk of atopic dermatitis during childhood and adolescence according to maternal rotating night shiftwork history before pregnancy, restricted to singleton, full-term births.

	History of rotating night shift work					
	Never worked rotating night shifts	<3 yrs	3–5 yrs	≥6 yrs	P trend	Ever worked rotating night shifts
Maternal report of child’s atopic dermatitis*						
			OR (95% CI)			
Cases/participants	190/1,683	157/1,507	133/1,132	57/491		347/3,130
Basic model ^a^	1 (reference)	0.90 (0.71; 1.13)	1.03 (0.80; 1.31)	1.04 (0.76; 1.43)	0.56	0.97 (0.80; 1.17)
MV model 1^b^	1 (reference)	0.91 (0.72; 1.15)	1.04 (0.81; 1.33)	1.04 (0.75; 1.44)	0.58	0.98 (0.80; 1.19)
MV model 2^c^	1 (reference)	0.90 (0.71; 1.14)	1.06 (0.82; 1.36)	1.06 (0.76; 1.47)	0.46	0.98 (0.80; 1.19)
Maternal report of child’s asthma*				** **	** **	
** **			OR (95% CI)			
Cases/participants	248/1,683	245/1,507	170/1,132	87/491		502/3,130
Basic model ^a^	1 (reference)	1.12 (0.92; 1.37)	1.02 (0.82; 1.27)	1.24 (0.94; 1.62)	0.40	1.10 (0.93; 1.31)
MV model 1^b^	1 (reference)	1.12 (0.91; 1.36)	1.00 (0.81; 1.25)	1.22 (0.92; 1.61)	0.51	1.09 (0.92; 1.29)
MV model 2^c^	1 (reference)	1.10 (0.90; 1.36)	1.01 (0.81; 1.27)	1.27 (0.95; 1.70)	0.37	1.09 (0.92; 1.31)
Maternal report of child’s hay fever*			** **	** **	** **	
			OR (95% CI)			
Cases/participants	274/1,683	264/1,507	178/1,132	85/491		527/3,130
Basic model ^a^	1 (reference)	1.10 (0.91; 1.34)	0.96 (0.78; 1.19)	1.07 (0.81; 1.40)	0.82	1.05 (0.89; 1.23)
MV model 1^b^	1 (reference)	1.11 (0.92; 1.35)	0.96 (0.77; 1.19)	1.11 (0.84; 1.46)	0.96	1.06 (0.89; 1.25)
MV model 2^c^	1 (reference)	1.10 (0.89; 1.35)	0.99 (0.78; 1.25)	1.16 (0.86; 1.57)	0.74	1.07 (0.89; 1.28)

*Assessed in 2009 from GUTS Mothers’ questionnaire; Defined as physician-diagnosed eczema (atopic dermatitis), asthma, hay fever

Abbreviations: CI, confidence interval; OR, odds ratio; MV, multivariable model

^a^ Adjusted for offspring gender (boy/girl) and offspring age at GUTS baseline 2004

^**b**^ Additionally adjusted for maternal age at pregnancy, smoking status before pregnancy (never, current, past), alternative healthy eating score (quintiles), physical activity (METs hours/week; quintiles), husband’s education (less than 2yr college, 4yr college, grad school), parity (nulliparity, 1, 2, 3+ previous pregnancies), BMI before pregnancy (<25, 25–29, ≥30 kg/m^2^), geographic region of residence (West, Midwest (reference), South, Northeast) and Census tract education rate in 1989

^c^ Additionally adjusted for parental diagnosis of eczema, asthma and hay fever (yes/no)

Results remained unchanged when using children’s self-reported outcomes rather than maternal-reported outcomes (S1 Table). The proportion of mothers reporting atopic disease when their offspring did not were variable for the different outcomes (AD = 0.52; asthma = 0.22; hay fever = 0.67). We used consistent maternal-reported and child’s self-reported outcomes to present a more stringent outcome and though results slightly differed, they remained non-significant (AD OR_ever nightshifts_ = 1.19; 95% CI = 0.90–1.57; asthma OR_ever nightshifts_ = 1.06; 95% CI = 0.87–1.29; hay fever OR_ever nightshifts_ = 1.24; 95% CI = 0.93–1.64) (Table 3). S2 Table shows the associations between shift work and maternal reports of their offspring being diagnosed with all three atopic diseases (OR_ever nightshifts_ = 0.95; 95% CI = 0.60–1.49). S3 Table shows outcomes if mothers reported their child being diagnosed with either AD, asthma or hay fever. Although any report of night shift work was not associated with atopy risk (OR_ever nightshifts_ = 1.13; 95% CI = 0.97–1.31), we observed a suggestive trend of increased risk of atopy in offspring associated with longer duration of maternal night shift work (OR_≥6yrs night work_ = 1.27; 95% CI = 0.99–1.63; P_trend_ = 0.07).

**Table 3:** Consistent mother and child outcomes: Adjusted odds ratios (OR) for offspring risk of atopic dermatitis during childhood and adolescence according to maternal rotating night shiftwork history before pregnancy, restricted to singleton, full-term births.

	History of rotating night shift work					
	Never worked rotating night shifts	<3 yrs	3–5 yrs	≥6 yrs	P trend	Ever worked rotating night shifts
Mother’s report* and child’s self-reported atopic dermatitis**						
			OR (95% CI)			
Cases/participants	80/1,683	85/1,507	68/1,132	26/491		179/3,130
Basic model ^a^	1 (reference)	1.17 (0.85; 1.61)	1.26 (0.90; 1.77)	1.10 (0.70; 1.73)	0.28	1.19 (0.91; 1.57)
MV model 1^b^	1 (reference)	1.18 (0.86; 1.63)	1.24 (0.89; 1.74)	1.04 (0.66; 1.65)	0.46	1.18 (0.90; 1.56)
MV model 2^c^	1 (reference)	1.17 (0.85; 1.62)	1.26 (0.90; 1.77)	1.06 (0.67; 1.68)	0.39	1.19 (0.90; 1.57)
Mother’s report* and child’s self-reported asthma**				** **	** **	
** **			OR (95% CI)			
Cases/participants	197/1,683	190/1,507	131/1,132	69/491		390/3,130
Basic model ^a^	1 (reference)	1.09 (0.88; 1.35)	0.99 (0.78; 1.25)	1.23 (0.91; 1.65)	0.52	1.07 (0.89; 1.29)
MV model 1^b^	1 (reference)	1.08 (0.87; 1.35)	0.97 (0.76; 1.23)	1.18 (0.87; 1.61)	0.73	1.06 (0.87; 1.28)
MV model 2^c^	1 (reference)	1.07 (0.85; 1.34)	0.98 (0.76; 1.25)	1.23 (0.89; 1.70)	0.57	1.06 (0.87; 1.29)
Mother’s report* and child’s self-reported hay fever**			** **	** **	** **	
			OR (95% CI)			
Cases/participants	83/1,683	99/1,507	60/1,132	26/491		185/3,130
Basic model ^a^	1 (reference)	1.36 (1.00;1.84)	1.06 (0.75; 1.51)	1.06 (0.68; 1.67)	0.94	1.20 (0.92; 1.57)
MV model 1^b^	1 (reference)	1.38 (1.02; 1.88)	1.07 (0.75; 1.53)	1.12 (0.70; 1.78)	0.96	1.23 (0.93; 1.62)
MV model 2^c^	1 (reference)	1.36 (0.99; 1.86)	1.11 (0.77; 1.61)	1.13 (0.71; 1.82)	0.79	1.24 (0.93; 1.64)

* Assessed in 2009 GUTS Mothers’ questionnaire; Defined as physician-diagnosed eczema (atopic dermatitis), asthma, hay fever

**Assessed in 2006, 2008, 2013 from GUTS questionnaires; defined as physician-diagnosed eczema (atopic dermatitis), asthma, hay fever

Abbreviations: CI, confidence interval; OR, odds ratio; MV, multivariable model

^a^ Adjusted for offspring gender (boy/girl) and offspring age at GUTS baseline 2004

^**b**^ Additionally adjusted for maternal age at pregnancy, smoking status before pregnancy (never, current, past), alternative healthy eating score (quintiles), physical activity (METs hours/week; quintiles), husband’s education (less than 2yr college, 4yr college, grad school), parity (nulliparity, 1, 2, 3+ previous pregnancies), BMI before pregnancy (<25, 25–29, ≥30 kg/m^2^), geographic region of residence (West, Midwest (reference), South, Northeast) and Census tract education rate in 1989

^c^ Additionally adjusted for parental diagnosis of eczema, asthma and hay fever (yes/no)

We conducted stratified analyses by history of parental atopy (S4 Table) and maternal chronotype (S5 Table). While we observed some statistically significant findings, overall trends were inconsistent and no significant interaction was seen with increasing duration of night shift work (parental atopy P_interaction_ = 0.06; maternal chronotype P_interaction_ = 0.12).

### Maternal rotating night shift work during pregnancy

Out of the 545 mother-child pairs included in this analysis, 20% of mothers ever worked night shifts during pregnancy. The association between maternal night shift work during pregnancy and risk of maternal-reported offspring AD was not significant (MV model 2, OR_ever nighshifts_ = 0.60; 95% CI = 0.30–1.20), neither did we observe any associations with risk of asthma (MV model 2, OR_ever nightshifts_ = 0.61; 95% CI = 0.28–1.29) or hay fever (MV model 2 OR_ever nightshifts_ = 0.80; 95% CI = 0.39–1.65) (see S6 Table).

## Discussion

Overall, our study involving mothers from the NHSII cohort and their offspring in GUTS found no apparent association between maternal history of night shift work before pregnancy or during pregnancy and AD or other atopic diseases in their offspring. While a few associations were statistically significant in our stratified models, they were inconsistent and did not present any clear patterns of association.

Night shift work, a major source of chronic circadian disruption, has previously been linked to increased risk of chronic diseases [24,43–45] and has also been suggested as a biological, psychosocial and physiological health stressor [46,47] due to the substantial changes in circadian rhythm that night workers encounter. Circadian rhythm plays a role in the pathogenesis of allergic reactions and is important for regulating specific immune functions [48,49]. This suggests that the stress induced by changes in the circadian clock might have an impact on developing AD or other related diseases.

An animal study by Takita et al. investigated whether biological clock dysfunction affected the pathogenesis of contact hypersensitivity (CHS) in *CLOCK* mutant mice which elicits a similar response to human allergic contact dermatitis. They discovered that circadian dysfunction in CLOCK mutant mice impacted their allergic response exhibited by increased ear swelling after 24 hours, increased serum IgE concentration and increased number of mast cells when compared to wild-type mice [50]. This provides support for the hypothesis that disruptions in the circadian clock give way to the onset of allergic diseases.

Lin et al. found that—in humans—psychosocial stress in the mother is a potential risk factor for elevated cord blood IgE and hence in the newborn [36]. Immunoglobulin E is reportedly strongly associated with atopic multi-morbidities such as eczema, asthma and rhinitis, throughout childhood and adolescence within the individual [51], suggesting that not only lifestyle factors and behaviors during childhood influence the development of allergies, but that in utero exposures might also be important.

Maternal stress caused from different factors such as postnatal psychological problems [35], prenatal depression/anxiety [52,53] and employment/job strain [54,55] is shown to increase the risk of eczema and asthma in their offspring. A review by Chan et al. also examined studies addressing similar maternal stress factors and found postpartum and prenatal depression, anxiety and adverse life events to be associated with an increased risk of developing AD in their offspring '[56], but to our knowledge, there is no other study that has examined the relationship specifically between maternal night shift work exposure before and during pregnancy and AD.

Strengths of our study include longitudinal follow-up of detailed information of mothers and offspring as well as being able to consider multiple potential confounding variables. Our study has a few limitations that should be mentioned. Regarding night shift work during pregnancy, the timing of the supplemental pregnancy questionnaire reduced the time window (1993–1995) to obtain during-pregnancy exposure information. Consequently, we had limited power when assessing the relationship between during-pregnancy shift work exposure and atopic outcomes. Furthermore, our study relied solely on self-reported outcomes for atopic diseases, which could contribute to outcome misclassifications. Diagnoses for AD in GUTS have not been validated, however, self and caregiver-reported childhood AD has previously shown to be valid [57]. Unfortunately, we lack information regarding the age of diagnosis of AD, asthma, or hay fever. Since GUTS participants were aged 11 to 17 when they were first asked if they had ever been diagnosed with AD, this constitutes another possibility for outcome misclassification; however, in secondary analyses restricting to consistent self-report of the outcome by both the mother and the child, results remained largely unchanged. In previous studies, the most important factors associated with correctly remembering and reporting AD included long duration of AD in childhood, concomitant atopic disease, and eczema after the age of 15 [58,59]. Considering such a scenario in our study, we might have missed any associations between rotating night shift work of the mothers before and during pregnancy with less severe AD in their offspring, which appears unlikely if there is no association with presumably more severe/longer duration AD to begin with. Further, we only had limited information on paternal factors. Nevertheless, we could adjust for husband’s education as a proxy for socioeconomic status in all our multivariable models, which we considered the most important confounding variable possibly affecting both maternal shift work exposure and offspring weight outcomes. Lastly, since mothers in our analyses were slightly older than the nation’s average, these findings may not be generalizable to younger mothers.

In conclusion, we found no consistent associations between maternal night shift work and risk for atopic conditions in offspring. Future studies with more detailed exposure information and specific age at disease diagnosis would be helpful to examine this relationship in more detail.

## Supporting information

S1 Fig Flow chart showing how we derived the analysis sample from the full GUTS 2 cohort. (DOCX)

S1 Table Child’s self-reported atopic dermatitis, asthma and hay fever: Adjusted odds ratios (OR) and 95% confidence intervals (CI) for offspring atopic dermatitis during childhood and adolescence according to maternal rotating night shiftwork history before pregnancy, restricted to singleton, full-term births. (DOCX)

S2 Table All allergies outcome: Adjusted odds ratios (OR) and 95% confidence intervals (CI) for offspring atopic dermatitis during childhood and adolescence according to maternal rotating night shiftwork history before pregnancy, restricted to singleton, full-term births. (DOCX)

S3 Table Any allergy outcome: Adjusted odds ratios (OR) and 95% confidence intervals (CI) for offspring atopic dermatitis during childhood and adolescence according to maternal rotating night shiftwork history before pregnancy, restricted to singleton, full-term births. (DOCX)

S4 Table Adjusted odds ratios (OR) and 95% confidence intervals (CI) for offspring atopic dermatitis, asthma and hay fever during childhood and adolescence according to maternal rotating night shiftwork history before pregnancy, restricted to singleton, full-term births, stratified by parental atopy^x^. (DOCX)

S5 Table Adjusted odds ratios (OR) and 95% confidence intervals (CI) for offspring atopic dermatitis, asthma and hay fever during childhood and adolescence according to maternal rotating night shiftwork history before pregnancy, restricted to singleton, full-term births, stratified by maternal chronotype (Definite morning type vs. Intermediate type vs. Definite evening type). (DOCX)

S6 Table Adjusted odds ratios (OR) and 95% confidence intervals (CI) for offspring atopic dermatitis, asthma and hay fever during childhood and adolescence by night shift exposure during pregnancy, restricted to singleton, full-term births. (DOCX)
