# Supplementary material for: Night shift work surrounding pregnancy and offspring risk of atopic disease
Source: PLoS One. 2020 Apr 16;15(4):e0231784. doi: 10.1371/journal.pone.0231784 (PMC7161965; doi:10.1371/journal.pone.0231784)

**Supplemental Figure 1. Flow chart showing how we derived the analysis sample from the full GUTS 2 cohort.**


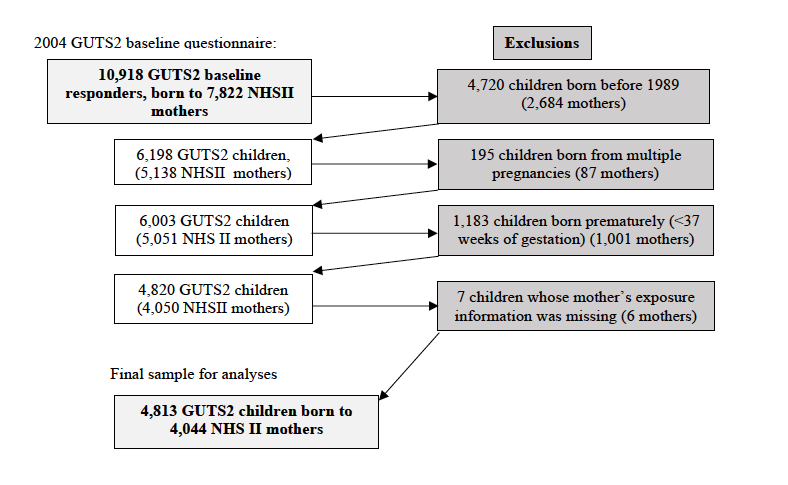

Supplement: S1 Fig — (DOCX) [file pone.0231784.s001.docx]
