# Supplementary material for: Night shift work surrounding pregnancy and offspring risk of atopic disease
Source: PLoS One. 2020 Apr 16;15(4):e0231784. doi: 10.1371/journal.pone.0231784 (PMC7161965; doi:10.1371/journal.pone.0231784)
Supplement: S1 Table — (DOCX) [file pone.0231784.s002.docx]

**Supplemental Table 1. Child’s self-reported atopic dermatitis, asthma and hay fever: Adjusted odds ratios (OR) and 95% confidence intervals (CI) for offspring atopic dermatitis during childhood and adolescence according to maternal rotating night shiftwork history before pregnancy, restricted to singleton, full-term births**

|  | **History of rotating night shift work** | | | | | |
| --- | --- | --- | --- | --- | --- | --- |
|  | **Never worked rotating night shifts** | **<3 yrs** | **3-5 yrs** | **≥6 yrs** | **P trend** | **Ever worked rotating night shifts** |
| **Child’s self-reported atopic dermatitis*** | | |  |  |  |  |
|  |  |  | OR (95 % CI) |  |  |  |
| Cases/participants | 130/1,683 | 131/1,507 | 106/1,132 | 40/491 |  | 277/3,130 |
| Basic model ^a^ | 1 (reference) | 1.11 (0.86; 1.43) | 1.23 (0.93; 1.62) | 1.06 (0.73; 1.53) | 0.27 | 1.14 (0.92; 1.42) |
| MV model 1^b^ | 1 (reference) | 1.12 (0.87; 1.45) | 1.21 (0.92; 1.59) | 1.02 (0.70; 1.49) | 0.42 | 1.14 (0.91; 1.42) |
| MV model 2^c^ | 1 (reference) | 1.11 (0.86; 1.44) | 1.22 (0.92; 1.60) | 1.03 (0.71; 1.51) | 0.37 | 1.14 (0.91; 1.42) |
| **Child’s self-reported asthma*** | | | |  |  |  |
|  |  |  | OR (95 % CI) |  |  |  |
| Cases/participants | 280/1,683 | 265/1,507 | 202/1,132 | 91/491 |  | 558/3,130 |
| Basic model ^a^ | 1 (reference) | 1.06 (0.88; 1.28) | 1.09 (0.89; 1.33) | 1.12 (0.86; 1.47) | 0.33 | 1.08 (0.92; 1.27) |
| MV model 1^b^ | 1 (reference) | 1.08 (0.89; 1.31) | 1.09 (0.89; 1.34) | 1.12 (0.85; 1.48) | 0.36 | 1.09 (0.93; 1.29) |
| MV model 2^c^ | 1 (reference) | 1.07 (0.88; 1.30) | 1.10 (0.89; 1.36) | 1.15 (0.87; 1.52) | 0.27 | 1.09 (0.93; 1.29) |
| **Child’s self-reported hay fever*** | | |  |  |  |  |
|  |  |  | OR (95 % CI) |  |  |  |
| Cases/participants | 141/1,683 | 151/1,507 | 104/1,132 | 40/491 |  | 295/3,130 |
| Basic model ^a^ | 1 (reference) | 1.21 (0.95; 1.55) | 1.09 (0.83; 1.43) | 0.97 (0.67; 1.39) | 0.97 | 1.13 (0.91; 1.39) |
| MV model 1^b^ | 1 (reference) | 1.25 (0.97; 1.60) | 1.12 (0.85; 1.47) | 1.00 (0.69; 1.46) | 0.88 | 1.16 (0.94; 1.45) |
| MV model 2^c^ | 1 (reference) | 1.24 (0.97; 1.59) | 1.15 (0.87; 1.52) | 1.02 (0.69; 1.49) | 0.72 | 1.18 (0.94; 1.47) |

* Assessed in 2006, 2008, 2013 from GUTS questionnaires; defined as physician-diagnosed eczema (atopic dermatitis), asthma, hay fever

Abbreviations: CI, confidence interval; OR, odds ratio; MV, multivariable model

^a^ Adjusted for offspring gender (boy/girl) and offspring age at GUTS baseline 2004

**^b^** Additionally adjusted for maternal age at pregnancy, smoking status before pregnancy (never, current, past), alternative healthy eating score (quintiles), physical activity (METs hours/week; quintiles), husband’s education (less than 2yr college, 4yr college, grad school), parity (nulliparity, 1, 2, 3+ previous pregnancies), BMI before pregnancy (<25, 25-29, ≥30 kg/m^2^), geographic region of residence ( West, Midwest (reference), South, Northeast) and Census tract education rate in 1989

^c^ Additionally adjusted for parental diagnosis of eczema, asthma and hay fever (yes/no)
