# Supplementary material for: Night shift work surrounding pregnancy and offspring risk of atopic disease
Source: PLoS One. 2020 Apr 16;15(4):e0231784. doi: 10.1371/journal.pone.0231784 (PMC7161965; doi:10.1371/journal.pone.0231784)
Supplement: S2 Table — (DOCX) [file pone.0231784.s003.docx]

**Supplemental Table 2. All allergies outcome: Adjusted odds ratios (OR) and 95% confidence intervals (CI) for offspring atopic dermatitis during childhood and adolescence according to maternal rotating night shiftwork history before pregnancy, restricted to singleton, full-term births**

|  | **History of rotating night shift work** | | | | | |
| --- | --- | --- | --- | --- | --- | --- |
|  | **Never worked rotating night shifts** | **<3 yrs** | **3-5 yrs** | **≥6 yrs** | **P trend** | **Ever worked rotating night shifts** |
| **Mother’s report of child’s atopic dermatitis AND asthma AND hay fever*** | | |  |  |  |  |
|  |  |  | OR (95 % CI) |  |  |  |
| Cases/participants | 34/1,683 | 32/1,507 | 15/1,132 | 10/491 |  | 57/3,130 |
| Basic model ^a^ | 1 (reference) | 1.11 (0.68; 1.83) | 0.68 (0.36; 1.28) | 1.08 (0.53; 2.22) | 0.44 | 0.95 (0.61; 1.48) |
| MV model 1^b^ | 1 (reference) | 1.19 (0.72; 1.98) | 0.69 (0.37; 1.31) | 1.13 (0.54; 2.35) | 0.48 | 1.00 (0.64; 1.57) |
| MV model 2^c^ | 1 (reference) | 1.09 (0.66; 1.82) | 0.68 (0.36; 1.29) | 1.11 (0.53; 2.34) | 0.49 | 0.95 (0.60; 1.49) |

*Assessed in 2009 from the GUTS Mothers’ Questionnaire; Defined as physician-diagnosed eczema (atopic dermatitis), asthma and hay fever

Abbreviations: CI, confidence interval; OR, odds ratio; MV, multivariable model

^a^ Adjusted for offspring gender (boy/girl) and offspring age at GUTS baseline 2004

**^b^** Additionally adjusted for maternal age at pregnancy, smoking status before pregnancy (never, current, past), alternative healthy eating score (quintiles), physical activity (METs hours/week; quintiles), husband’s education (less than 2yr college, 4yr college, grad school), parity (nulliparity, 1, 2, 3+ previous pregnancies), BMI before pregnancy (<25, 25-29, ≥30 kg/m^2^), geographic region of residence ( West, Midwest (reference), South, Northeast) and Census tract education rate in 1989

^c^ Additionally adjusted for parental diagnosis of eczema, asthma and hay fever (yes/no)
