# Supplementary material for: Night shift work surrounding pregnancy and offspring risk of atopic disease
Source: PLoS One. 2020 Apr 16;15(4):e0231784. doi: 10.1371/journal.pone.0231784 (PMC7161965; doi:10.1371/journal.pone.0231784)
Supplement: S3 Table — (DOCX) [file pone.0231784.s004.docx]

**Supplemental Table 3. Any allergy outcome: Adjusted odds ratios (OR) and 95% confidence intervals (CI) for offspring atopic dermatitis during childhood and adolescence according to maternal rotating night shiftwork history before pregnancy, restricted to singleton, full-term births**

|  | **History of rotating night shift work** | | | | | |
| --- | --- | --- | --- | --- | --- | --- |
|  | **Never worked rotating night shifts** | **<3 yrs** | **3-5 yrs** | **≥6 yrs** | **P trend** | **Ever worked rotating night shifts** |
| **Mother’s report of child’s atopic dermatitis OR asthma OR hay fever*** | | |  |  |  |  |
|  |  |  | OR (95 % CI) |  |  |  |
| Cases/participants | 510/1,683 | 489/1,507 | 365/1,132 | 168/491 |  | 1,022/3,130 |
| Basic model ^a^ | 1 (reference) | 1.10 (0.94; 1.28) | 1.08 (0.91; 1.28) | 1.17 (0.94; 1.46) | 0.19 | 1.10 (0.97; 1.26) |
| MV model 1^b^ | 1 (reference) | 1.09 (0.94; 1.28) | 1.07 (0.90; 1.27) | 1.18 (0.94; 1.47) | 0.23 | 1.10 (0.96; 1.26) |
| MV model 2^c^ | 1 (reference) | 1.09 (0.92; 1.30) | 1.12 (0.93; 1.35) | 1.27 (0.99; 1.63) | 0.07 | 1.13 (0.97; 1.31) |

*Assessed in 2009 from the GUTS Mothers’ Questionnaire; Defined as physician-diagnosed eczema (atopic dermatitis), asthma and hay fever

Abbreviations: CI, confidence interval; OR, odds ratio; MV, multivariable model

^a^ Adjusted for offspring gender (boy/girl) and offspring age at GUTS baseline 2004

**^b^** Additionally adjusted for maternal age at pregnancy, smoking status before pregnancy (never, current, past), alternative healthy eating score (quintiles), physical activity (METs hours/week; quintiles), husband’s education (less than 2yr college, 4yr college, grad school), parity (nulliparity, 1, 2, 3+ previous pregnancies), BMI before pregnancy (<25, 25-29, ≥30 kg/m^2^), geographic region of residence ( West, Midwest (reference), South, Northeast) and Census tract education rate in 1989

^c^ Additionally adjusted for parental diagnosis of eczema, asthma and hay fever (yes/no)
