# Supplementary material for: Night shift work surrounding pregnancy and offspring risk of atopic disease
Source: PLoS One. 2020 Apr 16;15(4):e0231784. doi: 10.1371/journal.pone.0231784 (PMC7161965; doi:10.1371/journal.pone.0231784)
Supplement: S4 Table — (DOCX) [file pone.0231784.s005.docx]

**Supplemental Table 4. Adjusted odds ratios (OR) and 95% confidence intervals (CI) for offspring atopic dermatitis, asthma and hay fever during childhood and adolescence according to maternal rotating night shiftwork history before pregnancy, restricted to singleton, full-term births, stratified by parental atopy^x^**

|  | **History of rotating night shift work** | | | | | |
| --- | --- | --- | --- | --- | --- | --- |
|  | **Never worked rotating night shifts** | **<3 yrs** | **3-5 yrs** | **≥6 yrs** | **P trend** | **Ever worked rotating night shifts** |
| **Maternal report of child’s atopic dermatitis*** | | | | | |  |
| **Parental atopy** | | | | | |  |
|  |  |  | OR (95 % CI) |  |  |  |
| Cases/participants | 148/752 | 103/688 | 100/499 | 40/212 |  | 243/1,399 |
| Basic model ^a^ | 1 (reference) | 0.71 (0.54; 0.94) | 1.02 (0.76; 1.36) | 0.97 (0.65; 1,43) | 0.63 | 0.85 (0.68; 1.08) |
| MV model 1^b^ | 1 (reference) | 0.73 (0.55; 0.97) | 1.05 (0.78; 1.41) | 0.98 (0.65; 1.46) | 0.53 | 0.87 (0.69; 1.11) |
| **No parental atopy** | |  |  |  |  |  |
|  |  |  | OR (95 % CI) |  |  |  |
| Cases/participants | 42/931 | 54/819 | 33/633 | 17/279 |  | 104/1,731 |
| Basic model ^a^ | 1 (reference) | 1.45 (0.95; 2.22) | 1.13 (0.70; 1.82) | 1.35 (0.76; 2.40) | 0.59 | 1.31 (0.90; 1.92) |
| MV model 1^b^ | 1 (reference) | 1.47 (0.96; 2.25) | 1.09 (0.68; 1.76) | 1.35 (0.76; 2.40) | 0.67 | 1.31 (0.90; 1.90) |
|  |  |  |  | P (Interaction) =0.50 | |  |
| **Maternal report of child’s asthma*** | | | |  |  |  |
| **Parental atopy** |  |  |  |  |  |  |
|  |  |  | OR (95 % CI) |  |  |  |
| Cases/participants | 200/752 | 186/688 | 122/499 | 63/212 |  | 371/1,399 |
| Basic model ^a^ | 1 (reference) | 1.01 (0.80; 1.29) | 0.89 (0.69; 1.17) | 1.17 (0.83; 1.64) | 0.99 | 0.99 (0.81; 1.22) |
| MV model 1^b^ | 1 (reference) | 0.98 (0.77; 1.25) | 0.86 (0.65; 1.12) | 1.11 (0.78; 1.58) | 0.71 | 0.95 (0.77; 1.17) |
| **No parental atopy** |  |  |  |  |  |  |
|  |  |  | OR (95 % CI) |  |  |  |
| Cases/participants | 48/931 | 59/819 | 48/633 | 24/279 |  | 131/1,731 |
| Basic model ^a^ | 1 (reference) | 1.42 (0.95; 2.14) | 1.49 (0.97; 2.29) | 1.70 (1.01; 2.85) | 0.03 | 1.49 (1.05; 2.12) |
| MV model 1^b^ | 1 (reference) | 1.38 (0.92; 2.09) | 1.52 (0.99; 2.33) | 1.88 (1.10; 3.21) | 0.02 | 1.50 (1.05; 2.15) |
|  |  |  |  | P (Interaction) =0.06 |  |  |
| **Maternal report of child’s hay fever*** |  |  |  |  |  |  |
| **Parental atopy** |  |  |  |  |  |  |
|  |  |  | OR (95 % CI) |  |  |  |
| Cases/participants | 242/752 | 230/688 | 146/499 | 72/212 |  | 448/1,399 |
| Basic model ^a^ | 1 (reference) | 1.07 (0.85; 1.34) | 0.87 (0.68; 1.13) | 1.09 (0.79; 1.50) | 0.59 | 1.00 (0.82; 1.21) |
| MV model 1^b^ | 1 (reference) | 1.07 (0.85; 1.34) | 0.89 (0.69; 1.16) | 1.12 (0.80; 1.56) | 0.76 | 1.01 (0.83; 1.23) |
| **No parental atopy** |  |  |  |  |  |  |
|  |  |  | OR (95 % CI) |  |  |  |
| Cases/participants | 32/931 | 34/819 | 32/633 | 13/279 |  | 79/1,731 |
| Basic model ^a^ | 1 (reference) | 1.28 (0.77; 2.14) | 1.56 (0.93; 2.62) | 1.37 (0.68; 2.76) | 0.12 | 1.40 (0.90; 2.17) |
| MV model 1^b^ | 1 (reference) | 1.30 (0.77; 2.18) | 1.48 (0.87; 2.50) | 1.48 (0.72; 3.03) | 0.14 | 1.39 (0.89; 2.17) |
|  |  |  |  | P (Interaction) =0.18 |  |  |

**^x^** Defined as yes/no diagnosis of ever having any eczema (atopic dermatitis) or asthma or hay fever in either the mother or father.

*Assessed in 2009 from GUTS Mothers’ Questionnaire; Defined as physician-diagnosed eczema (atopic dermatitis), asthma, hay fever

Abbreviations: CI, confidence interval; OR, odds ratio; MV, multivariable model

^a^ Adjusted for offspring gender (boy/girl) and offspring age at GUTS baseline 2004

**^b^** Additionally adjusted for maternal age at pregnancy, smoking status before pregnancy (never, current, past), alternative healthy eating score (quintiles), physical activity (METs hours/week; quintiles), husband’s education (less than 2yr college, 4yr college, grad school), parity (nulliparity, 1, 2, 3+ previous pregnancies), BMI before pregnancy (<25, 25-29, ≥30 kg/m^2^), geographic region of residence ( West, Midwest (reference), South, Northeast) and Census tract education rate in 1989
