# Supplementary material for: Night shift work surrounding pregnancy and offspring risk of atopic disease
Source: PLoS One. 2020 Apr 16;15(4):e0231784. doi: 10.1371/journal.pone.0231784 (PMC7161965; doi:10.1371/journal.pone.0231784)
Supplement: S5 Table — (DOCX) [file pone.0231784.s006.docx]

**Supplemental Table 5. Adjusted odds ratios (OR) and 95% confidence intervals (CI) for offspring atopic dermatitis, asthma and hay fever during childhood and adolescence according to maternal rotating night shiftwork history before pregnancy, restricted to singleton, full-term births, stratified by maternal chronotype (Definite morning type vs. Intermediate type vs. Definite evening type)**

|  | **History of rotating night shift work** | | | | | |
| --- | --- | --- | --- | --- | --- | --- |
|  | **Never worked rotating night shifts** | **<3 yrs** | **3-5 yrs** | **≥6 yrs** | **P trend** | **Ever worked rotating night shifts** |
| **Maternal report of child’s atopic dermatitis*** | | | | | |  |
| **Definite morning types** | | | | | |  |
|  |  |  | OR (95 % CI) |  |  |  |
| Cases/participants | 58/542 | 60/528 | 39/391 | 18/182 |  | 117/1,101 |
| Basic model ^a^ | 1 (reference) | 1.05 (0.71; 1.54) | 0.91 (0.59; 1.42) | 0.90 (0.51; 1.59) | 0.55 | 0.97 (0.69; 1.37) |
| MV model 1^b^ | 1 (reference) | 1.06 (0.71; 1.59) | 0.87 (0.56; 1.36) | 0.88 (0.50; 1.56) | 0.43 | 0.96 (0.68; 1.37) |
| MV model 2^c^ | 1 (reference) | 1.03 (0.68; 1.56) | 0.83 (0.53; 1.31) | 0.85 (0.48; 1.50) | 0.34 | 0.93 (0.65; 1.33) |
| **Intermediate types** | |  |  |  |  |  |
|  |  |  | OR (95 % CI) |  |  |  |
| Cases/participants | 108/955 | 83/832 | 77/627 | 35/256 |  | 195/1,715 |
| Basic model ^a^ | 1 (reference) | 0.86 (0.63; 1.16) | 1.08 (0.78; 1.48) | 1.25 (0.83; 1.89) | 0.23 | 0.99 (0.77; 1.28) |
| MV model 1^b^ | 1 (reference) | 0.86 (0.63; 1.18) | 1.10 (0.79; 1.52) | 1.22 (0.79; 1.86) | 0.24 | 1.00 (0.77; 1.30) |
| MV model 2^c^ | 1 (reference) | 0.87 (0.63; 1.20) | 1.15 (0.83; 1.61) | 1.28 (0.83; 1.98) | 0.14 | 1.02 (0.78; 1.34) |
| **Definite evening types** |  |  |  |  | |  |
|  |  |  | OR (95 % CI) |  |  |  |
| Cases/participants | 24/183 | 14/141 | 16/113 | 4/53 |  | 34/307 |
| Basic model ^a^ | 1 (reference) | 0.76 (0.38; 1.53) | 1.05 (0.49; 2.28) | 0.70 (0.24; 2.05) | 0.85 | 0.85 (0.48; 1.52) |
| MV model 1^b^ | 1 (reference) | 0.73 (0.36; 1.50) | 0.90 (0.40; 1.99) | 0.69 (0.20; 2.30) | 0.62 | 0.78 (0.43; 1.41) |
| MV model 2^c^ | 1 (reference) | 0.69 (0.33; 1.45) | 0.98 (0.43; 2.22) | 0.67 (0.19; 2.37) | 0.74 | 0.78 (0.42; 1.44) |

P (Interaction) =0.22

| **Maternal report of child’s asthma*** | | | |  |  |  |
| --- | --- | --- | --- | --- | --- | --- |
| **Definite morning types** |  |  |  |  |  |  |
|  |  |  | OR (95 % CI) |  |  |  |
| Cases/participants | 72/542 | 86/528 | 62/391 | 36/182 |  | 184/1,101 |
| Basic model ^a^ | 1 (reference) | 1.26 (0.89; 1.77) | 1.22 (0.84; 1.78) | 1.58 (1.01; 2.47) | 0.09 | 1.30 (0.96; 1.75) |
| MV model 1^b^ | 1 (reference) | 1.25 (0.88; 1.78) | 1.23 (0.84; 1.81) | 1.51 (0.94; 2.42) | 0.14 | 1.28 (0.94; 1.75) |
| MV model 2^c^ | 1 (reference) | 1.20 (0.83; 1.73) | 1.18 (0.78; 1.76) | 1.48 (0.90; 2.43) | 0.20 | 1.23 (0.89; 1.70) |
| **Intermediate types** |  |  |  |  |  |  |
|  |  |  | OR (95 % CI) |  |  |  |
| Cases/participants | 154/955 | 126/832 | 93/627 | 39/256 |  | 258/1,715 |
| Basic model ^a^ | 1 (reference) | 0.94 (0.72; 1.22) | 0.90 (0.67; 1.20) | 0.95 (0.64; 1.39) | 0.56 | 0.92 (0.74; 1.16) |
| MV model 1^b^ | 1 (reference) | 0.91 (0.69; 1.19) | 0.88 (0.66; 1.17) | 0.92 (0.62; 1.38) | 0.47 | 0.90 (0.71; 1.13) |
| MV model 2^c^ | 1 (reference) | 0.90 (0.68; 1.19) | 0.90 (0.67; 1.22) | 0.96 (0.63; 1.47) | 0.66 | 0.91 (0.72; 1.16) |
| **Definite evening types** |  |  | OR (95 % CI) |  |  |  |
|  |  |  |  |  |  |  |
| Cases/participants | 22/183 | 29/141 | 15/113 | 12/53 |  | 56/307 |
| Basic model ^a^ | 1 (reference) | 1.84 (0.99; 3.41) | 1.13 (0.55; 2.31) | 2.05 (0.93; 4.51) | 0.33 | 1.60 (0.93; 2.75) |
| MV model 1^b^ | 1 (reference) | 1.70 (0.90; 3.22) | 1.16 (0.55; 2.44) | 2.14 (0.94; 4.88) | 0.28 | 1.56 (0.89; 2.73) |
| MV model 2^c^ | 1 (reference) | 2.03 (1.00; 4.15) | 1.27 (0.58; 2.75) | 2.79 (1.20; 6.45) | 0.14 | 1.82 (0.99; 3.33) |

P (Interaction) =0.21

| **Maternal report of child’s hay fever*** |  |  |  |  |  |  |
| --- | --- | --- | --- | --- | --- | --- |
| **Definite morning types** |  |  |  |  |  |  |
|  |  |  | OR (95 % CI) |  |  |  |
| Cases/participants | 69/542 | 94/528 | 67/391 | 32/182 |  | 193/1,101 |
| Basic model ^a^ | 1 (reference) | 1.48 (1.05; 2.09) | 1.40 (0.96; 2.04) | 1.44 (0.91; 2.27) | 0.11 | 1.45 (1.07; 1.96) |
| MV model 1^b^ | 1 (reference) | 1.45 (1.02; 2.06) | 1.42 (0.97; 2.09) | 1.55 (0.96; 2.48) | 0.06 | 1.46 (1.07; 1.99) |
| MV model 2^c^ | 1 (reference) | 1.36 (0.93; 2.00) | 1.41 (0.94; 2.13) | 1.53 (0.93; 2.52) | 0.08 | 1.40 (1.01; 1.96) |
| **Intermediate types** |  |  |  |  |  |  |
|  |  |  | OR (95 % CI) |  |  |  |
| Cases/participants | 172/955 | 137/832 | 86/627 | 47/256 |  | 270/1,715 |
| Basic model ^a^ | 1 (reference) | 0.92 (0.72; 1.19) | 0.74 (0.56; 0.99) | 1.03 (0.71; 1.49) | 0.26 | 0.87 (0.70; 1.08) |
| MV model 1^b^ | 1 (reference) | 0.94 (0.73; 1.22) | 0.73 (0.54; 0.99) | 1.06 (0.72; 1.57) | 0.27 | 0.88 (0.70; 1.10) |
| MV model 2^c^ | 1 (reference) | 0.93 (0.71; 1.23) | 0.75 (0.54; 1.03) | 1.15 (0.75; 1.78) | 0.49 | 0.89 (0.70; 1.14) |
| **Definite evening types** |  |  |  |  |  |  |
|  |  |  | OR (95 % CI) |  |  |  |
| Cases/participants | 33/183 | 31/141 | 24/113 | 6/53 |  | 61/307 |
| Basic model ^a^ | 1 (reference) | 1.19 (0.68; 2.07) | 1.06 (0.57; 1.96) | 0.48 (0.19; 1.21) | 0.30 | 1.00 (0.62; 1.63) |
| MV model 1^b^ | 1 (reference) | 1.19 (0.67; 2.14) | 1.03 (0.54; 1.96) | 0.54 (0.21; 1.38) | 0.39 | 1.02 (0.62; 1.70) |
| MV model 2^c^ | 1 (reference) | 1.28 (0.65; 2.51) | 1.12 (0.56; 2.23) | 0.61 (0.22; 1.64) | 0.61 | 1.10 (0.63; 1.91) |
|  |  |  |  | P (Interaction) =0.12 |  |  |

*Assessed in 2009 from the GUTS Mothers’ Questionnaire; Defined as physician-diagnosed eczema (atopic dermatitis), asthma, hay fever

Abbreviations: CI, confidence interval; OR, odds ratio; MV, multivariable model

^a^ Adjusted for offspring gender (boy/girl) and offspring age at GUTS baseline 2004

**^b^** Additionally adjusted for maternal age at pregnancy, smoking status before pregnancy (never, current, past), alternative healthy eating score (quintiles), physical activity (METs hours/week; quintiles), husband’s education (less than 2yr college, 4yr college, grad school), parity (nulliparity, 1, 2, 3+ previous pregnancies), BMI before pregnancy (<25, 25-29, ≥30 kg/m^2^), geographic region of residence ( West, Midwest (reference), South, Northeast) and Census tract education rate in 1989

^c^ Additionally adjusted for parental diagnosis of eczema, asthma and hay fever (yes/no)
