# Supplementary material for: Night shift work surrounding pregnancy and offspring risk of atopic disease
Source: PLoS One. 2020 Apr 16;15(4):e0231784. doi: 10.1371/journal.pone.0231784 (PMC7161965; doi:10.1371/journal.pone.0231784)
Supplement: S6 Table — (DOCX) [file pone.0231784.s007.docx]

**Supplemental Table 6. Adjusted odds ratios (OR) and 95% confidence intervals (CI) for offspring atopic dermatitis, asthma and hay fever during childhood and adolescence by night shift exposure during pregnancy, restricted to singleton, full-term births.**

|  | **Average number of night shifts/month during pregnancy** |  | |
| --- | --- | --- | --- |
|  | **None** | **Any no. nights/mo** | |
| **Maternal report of child’s atopic dermatitis*** | | |  |
|  |  | OR (95 % CI) | |
| Cases/participants | 55/435 | 10/110 | |
| Basic model ^a^ | 1 (reference) | 0.69 (0.34; 1.40) | |
| MV model 1^b^ | 1 (reference) | 0.61 (0.31; 1.22) | |
| MV model 2^c^ | 1 (reference) | 0.60 (0.30; 1.20) | |
| **Maternal report of child’s asthma*** | | | |
|  |  | OR (95 % CI) | |
| Cases/participants | 72/435 | 14/110 | |
| Basic model ^a^ | 1 (reference) | 0.73 (0.39; 1.36) | |
| MV model 1^b^ | 1 (reference) | 0.66 (0.33; 1.31) | |
| MV model 2^c^ | 1 (reference) | 0.61 (0.28; 1.29) | |
| **Maternal report of child’s hay fever*** |  |  | |
|  |  | OR (95 % CI) | |
| Cases/participants | 68/435 | 14/110 | |
| Basic model ^a^ | 1 (reference) | 0.77 (0.41;1.45) | |
| MV model 1^b^ | 1 (reference) | 0.83 (0.44; 1.58) | |
| MV model 2^c^ | 1 (reference) | 0.80 (0.39; 1.65) | |

*Assessed in 2009 from GUTS Mothers’ Questionnaire; Defined as physician-diagnosed eczema (atopic dermatitis), asthma, hay fever

Abbreviations: CI, confidence interval; OR, odds ratio; MV, multivariable model

^a^ Adjusted for offspring gender (boy/girl) and offspring age at GUTS baseline 2004

**^b^** Additionally adjusted for maternal age at pregnancy, smoking status before pregnancy (never, current, past), alternative healthy eating score (quintiles), physical activity (METs hours/week; quintiles), husband’s education (less than 2yr college, 4yr college, grad school), parity (nulliparity, 1, 2, 3+ previous pregnancies), BMI before pregnancy (<25, 25-29, ≥30 kg/m^2^), geographic region of residence ( west, midwest (reference), south, northeast) and Census tract education rate in 1989, history of rotating night shift work (never, <3 yrs, 3-5 yrs, ≥ 6 yrs)

^c^ Additionally adjusted for parental diagnosis of eczema, asthma and hay fever (yes/no)
